# Visual acuity improvement after phacoemulsification cataract surgery in patients aged ≥90 years

**DOI:** 10.1186/s12886-018-0950-8

**Published:** 2018-10-29

**Authors:** Taku Toyama, Takashi Ueta, Masato Yoshitani, Rei Sakata, Jiro Numaga

**Affiliations:** 1 Department of Ophthalmology, Tokyo Metropolitan Geriatric Hospital and Institute of Gerontology, 35-2, Sakae-cho, Itabashi-ku, Tokyo, 173-0015 Japan; 2 Department of Ophthalmology, Graduate School of Medicine and Faculty of Medicine, The University of Tokyo, 7-3-1, Hongo, Bunkyo-ku, Tokyo, 113-8655 Japan; 3 Department of Ophthalmology, Center Hospital of the National Center for Global Health and Medicine, 1-21-1 Toyama Shinjyuku-ku, Tokyo, 162-8655 Japan

**Keywords:** Cataract, Phacoemulsification, Visual acuity, Surgical outcomes

## Abstract

**Background:**

Visual acuity (VA) outcomes after phacoemulsification cataract surgery in the very elderly (≥90 years) compared to those in younger patients remain unclear till date.

**Methods:**

We retrospectively investigated 138 (group 1) and 152 (group 2) eyes in patients aged ≥90 and < 80 years, respectively, with senile cataracts who underwent phacoemulsification and intraocular lens implantation between 2014 and 2016. Four highly experienced ophthalmic surgeons performed the procedures. Intra- and post-operative complications were compared between the two groups. To investigate the effectiveness of cataract surgery in improving best-corrected VA (BCVA) at 1 and 3 months postoperatively, multiple regression analysis was performed with variables of age, cataract grades, sex, and history of diabetes mellitus (DM) and hypertension.

**Results:**

The intra- and post-operative complication rates were similar between the two groups. After adjusting for the difference in cataract grades, multiple regression analysis indicated that BCVA improvement was equally favorable in both groups at 1 and 3 months postoperatively but was less favorable in patients with a history of DM at 3 months postoperatively (*P* = 0.042).

**Conclusion:**

Phacoemulsification in patients aged ≥90 years improves VA as effectively and safely as it does in younger patients, at least when performed by experienced surgeons.

## Background

Senile cataract is the most common cause of visual acuity (VA) loss in the elderly population, and it also impairs the overall quality of life. Significant cataract is considered to affect approximately 50% people by their 70s and 100% by their 90s [1]. Impaired VA in the elderly is associated with decreased capability for daily activities, decreased social functioning, and limited life expectancy [2–5].

As healthcare systems have prevailed, and surgical devices and techniques have advanced, phacoemulsification has been performed safely in many elderly patients, such as those aged ≥90 years. However, it is true that more challenges exist in the cataract surgery for such patients including harder cataract, shallower anterior chamber, smaller pupil size, higher rates of exfoliation syndrome, weaker Zinn’s zonule, and insufficient cooperation during surgery [6]. Evidence regarding visual outcomes after cataract surgery in such elderly patients remains limited.

Therefore, we evaluated the VA outcomes of cataract surgery in patients aged ≥90 years compared to those in younger patients.

## Methods

### Patients

The institutional review board of Tokyo Metropolitan Geriatric Hospital and Institute of Gerontology approved this retrospective study (certificate approval number: R15–54). We searched records of patients aged ≥90 years who underwent cataract surgery between January 2014 and December 2016 at Tokyo Metropolitan Geriatric Hospital; we identified 232 eyes of 145 patients who underwent phacoemulsification. The cataracts had caused VA impairment with unacceptable glare, diplopia, and/or reduced quality of vision. Comprehensive ophthalmic examinations were conducted pre- and post-operatively; best-corrected VA (BCVA), intraocular pressure, slit-lamp microscopy, and dilated-pupil funduscopic examination. Lens status was recorded as the pictures of slit-lamp microscopy as well as the descriptions. Optical coherence tomography was conducted to evaluate macular edema/thickening when deemed necessary by the physician.

A patient was excluded because comprehensive ophthalmic examinations were not feasible owing to a significant decline in his cognitive function. Another patient was excluded because he had undergone combined trabeculotomy. Additionally, 14 eyes were excluded because of ocular comorbidities that affected VA, including age-related macular degeneration in 4, branch retinal vein occlusion (BRVO) in 4, central retinal vein occlusion (CRVO) in 3, corneal opacity in 2, and advanced glaucoma in 1. One eye per each patient was enrolled in this study. If both the eyes were eligible, the first operated eyes were enrolled. As a result, 138 eyes of 138 patients aged ≥90 years were included in the study (group 1).

During the same 3-year period, 2566 cataract surgeries were performed in patients aged < 80 years. Because the age group was the most predominant for senile cataract surgery, it was regarded as the control group for the purpose of this study. Data on 80 consecutive eyes in each year from 2014 to 2016 were retrieved for the control group (158 patents, 240 eyes). Six eyes were excluded because of ocular comorbidities that affected VA, including BRVO in 3, CRVO in 1, diabetic macular edema in 1, and amblyopia in 1. One eye per each patient was enrolled so that the control group (group 2) consisted of 152 eyes of 152 patients aged < 80 years.

### Cataract surgery

Phacoemulsification with intraocular lens (IOL) implantation was performed using topical anesthesia with or without additional sub-Tenon anesthesia. All operations were performed by four highly experienced attending ophthalmologists. The procedures were identical in all patients. Using the INFINITI Vision System (Alcon, Inc., Fort Worth, TX, USA), a small 2.4-mm incision was made followed by continuous tear capsulotomy, hydrodissection, phacoemulsification, and insertion of the IOL. Post operative treatment consisted of topical combination levofloxacin, betamethasone, and nepafenac eye drops for 4 weeks, followed by nepafenac eye drop for another 2 months. The SRK/T formula was used to calculate lens power. Five IOL models were used in group 1 (HOYA 255 [Hoya Surgical Optics, Chino Hills, CA, USA] in 48 eyes, Alcon MN60AC in one, Alcon SN60WF in 57, Alcon SN6AT in 11, and HOYA XY1 in 21), and four IOL models in group 2 (HOYA 255 in 88 eyes, Alcon SN60WF in 57, Alcon MN60AC in one, and Alcon SN6AT in six).

### Data extraction

BCVA improvement at 1 and 3 months postoperatively was the primary outcome measure in our study. Data on cataract grade, age, sex, baseline BCVA, medical history of diabetes mellitus (DM) and hypertension (HT), ocular comorbidities, and intra- and post-operative complications were extracted. Decimal VA values were converted into the logarithm of the minimum angle of resolution (logMAR) for analysis.

### Statistical analysis

Statistical analysis was performed using EZR software, version 1.37 (Saitama Medical Center, Jichi Medical University, Saitama, Japan) [7]. Univariate analysis for numeric and categorical data was performed with Student’s *t* test and Fisher’s exact test, respectively. Multivariate analyses to determine factors affecting postoperative VA and its improvement from preoperative VA were performed using a multiple regression model with standard least-squares. *P* < 0.05 was considered statistically significant.

## Results

Patient characteristics are shown in Table 1. Mean age ± standard deviation (SD) was 92.2 ± 2.3 (range, 90–101) and 72.3 ± 5.6 (range, 52–79) years in groups 1 and 2, respectively, and there were 40/98 and 54/98 male/female patients, respectively. The male/female balance was not statistically different between the groups (*P* = 0.26). A history of DM was noted in 16 (11.6%) and 30 (19.7%) patients, respectively (*P* = 0.076) and a history of HT in 92 (66.7%) and 67 (44.1%), respectively (*P* < 0.001).

**Table 1:** Characteristics of the enrolled eyes

	Group 1 (≥90 years old)	Group 2 (< 80 years old)	*P* Value
Eyes (patients)	138	152	
Age, Mean ± SD (range)	92.3 ± 2.3	72.3 ± 5.6	< 0.001^*^
Male/female	40/98	54/98	0.259
Systemic comorbidities			
DM, patients (%)	16(11.6%)	30(19.7%)	0.0761
HT, patients (%)	92(66.7%)	67(44.1%)	< 0.001^*^
LOCS III Grading (Nuclear cataract)			< 0.001^*^
1	0	0	
2	9	33	
3	63	88	
4	49	24	
5	10	4	
6	7	3	
LOCS III Grading (Cortical cataract)			0.164
1	27	12	
2	58	77	
3	45	57	
4	8	6	
LOCS III Grading (Posterior cataract)			0.192
1	100	100	
2	13	14	
3	14	21	
4	8	15	
5	3	2	
Preoperative BCVA, logMAR ± SD	0.70 ± 0.60	0.46 ± 0.50	< 0.001^*^
Postoperative BCVA, logMAR ± SD			
1 month	0.11 ± 0.13	−0.01 ± 0.12	< 0.001^*^
3 months	0.11 ± 0.12	− 0.01 ± 0.11	< 0.001^*^

*SD* standard deviation

^*^Statistically significant *P* values

**Fig. 1:**
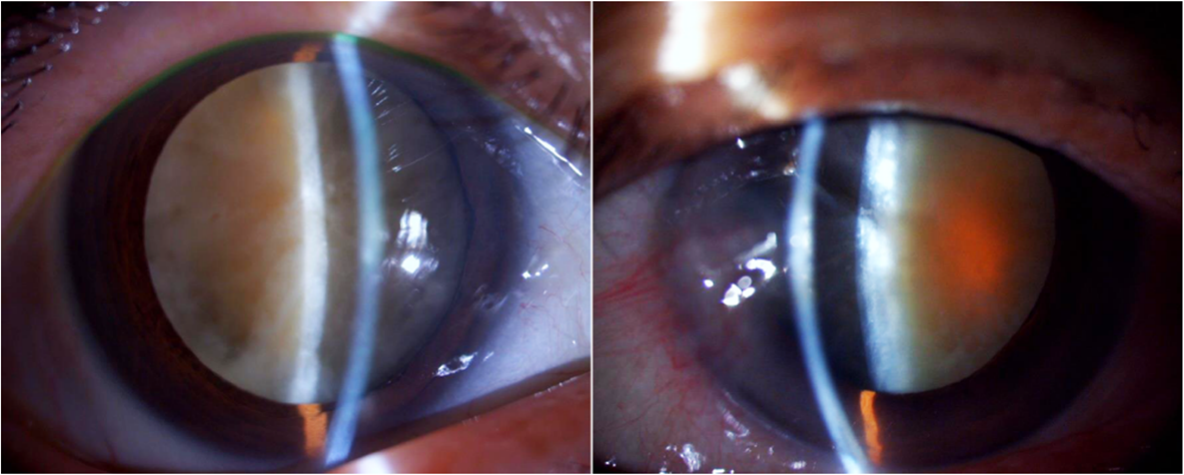
Representative pictures of higher grade cataracts in patients ≥90 years old

Severity of cataract was graded using the Lens Opacities Classification System III (LOCS III) cataract grade classification [8] (Table 1). As expected, the eyes in group 1 had more severe nuclear opacity compared to the eyes in group 2 (*P* < 0.001, Fig. 1). However, there was no significant difference in severity of cortical (*P* = 0.164) and posterior (*P* = 0.192) cataract between the groups (Table 1).

Preoperative BCVA (mean ± SD) was worse in group 1 than in group 2 (0.70 ± 0.60 vs. 0.40 ± 0.47 logMAR, respectively; *P* < 0.001; Table 1). Postoperative BCVA (mean ± SD) also was worse in group 1 than in group 2 at 1 and 3 months postoperatively (0.11 ± 0.13 vs. − 0.01 ± 0.12 and 0.11 ± 0.12 vs. − 0.01 ± 0.11 logMAR for 1 and 3 months, respectively; *P* < 0.001; Table 1).

Intraoperative complication rates were low in both groups (Table 2). In group 1, anterior capsular tear occurred in four eyes (2.9%), posterior capsule perforation in one (0.72%), and Zinn’s zonular dialysis in two (1.4%). In group 2, anterior capsular tear occurred in three eyes (2.0%), Zinn’s zonular dialysis in one (0.66%), and wound burning in one (0.66%). Anterior vitrectomy was necessary in one eye with posterior capsule perforation in group 1. In total, the intraoperative complication rate was not significantly different between groups 1 (5.0%) and 2 (3.3%; *P* = 0.56).

**Table 2:** Intraoperative complications

	Group 1 (≥90 years old)	Group 2 (< 80 years old)	*P* value
Anterior capsule tear	4 (2.9%)	3 (2.0%)	
Posterior capsule perforation	1 (0.72%)	0 (0%)	
Zinn’s zonular dialysis	2 (1.4%)	1 (0.66%)	
Wound burning	0 (0%)	1 (0.66%)	
Total	7 (5.0%)	5 (3.3%)	0.56

Postoperative complication rates are shown in Table 3. In group 1, IOP elevation requiring topical treatments occurred in three eyes (2.2%), corneal epithelial damage in three (2.2%), and cystoid macular edema in one (0.72%). In group 2, IOP elevation occurred in seven eyes (4.6%) and corneal epithelial damage in one (0.66%). One eye in group 2 needed yttrium-aluminum-garnet laser posterior capsulotomy by 3 months postoperatively (0.66%). Totally, the postoperative complication rate was not significantly different between groups 1 (5.1%) and 2 (5.9%; *P* = 0.80; Table 3).

**Table 3:** Postoperative complications

	Group 1 (≥90 years old)	Group 2 (< 80 years old)	*P* value
IOP elevation	3 (2.2%)	7 (4.6%)	
Corneal epithelial disorder	3 (2.2%)	1 (0.66%)	
PCO	0 (0%)	1 (0.66%)	
CME	1 (0.72%)	0 (0%)	
Total	7 (5.1%)	9 (5.9%)	0.80

*IOP* intraocular pressure, *PCO* posterior capsule opacity, *CME* cystoid macular edema

To understand the effect of phacoemulsification on VA in the different age groups, postoperative BCVA improvement was analyzed using a multiple regression model with explanatory variables comprising the different age groups, male/female balance, cataract grades, and history of DM and HT (Tables 4 and 5). As expected, higher grades in nuclear, cortical, and posterior cataracts were related to significantly larger BCVA improvement postoperatively (Tables 4 and 5). After adjusting for differences in cataract grades and other confounders, analysis showed that postoperative BCVA improvement in group 1 was as good as that in group 2 at 1 (Table 4) and 3 (Table 5) months. Male/female balance and history of HT did not have an effect. History of DM had no effect on BCVA improvement after 1 month (Table 4) but negatively affected BCVA improvements by 0.14 logMAR at 3 months postoperatively (*P* = 0.049; Table 5).

**Table 4:** Multiple regression analysis on variables potentially predicting BCVA improvement 1 month after surgery

	Estimate, logMAR (95%CI)	*P* value
Group 1 (≥90 years old)	−0.014 (− 0.099–0.072)	0.76
Male	−0.014 (− 0.099–0.072)	0.75
DM	− 0.034 (− 0.14–0.074)	0.54
HT	0.017 (− 0.063–0.098)	0.67
LOCS III (Nuclear Cataract, vs grade 2)		
3	0.19 (0.076–0.31)	0.0013^*^
4	0.37 (0.24–0.50)	< 0.001^*^
5	0.95 (0.74–1.2)	< 0.001^*^
6	2.0 (1.8–2.2)	< 0.001^*^
LOCS III (Cortical Cataract, vs grade 1)		
2	0.13 (0.0041–0.26)	0.043^*^
3	0.28 (0.15–0.41)	< 0.001^*^
4	0.65 (0.45–0.86)	< 0.001^*^
LOCS III (Posterior Cataract, vs grade 1)		
2	0.19 (0.046–0.33)	0.0096^*^
3	0.35 (0.23–0.47)	< 0.001^*^
4	0.74 (0.59–0.89)	< 0.001^*^
5	1.5 (1.1–1.8)	< 0.001^*^

*CI* confidence interval

^*^Statistically significant *P* value

**Table 5:** Multiple regression analysis on variables potentially predicting BCVA improvement 3 month after surgery

	Estimate, logMAR (95%CI)	*P* value
Group 1 (≥90 years old)	−0.060(− 0.17 to 0.048)	0.28
Male	0.039(−0.081 to 0.16)	0.52
DM	−0.14(− 0.27 to − 0.0053)	0.042^*^
HT	− 0.0087(− 0.11 to 0.095)	0.87
LOCS III (Nuclear Cataract, vs grade 2)		
3	0.25(0.095 to 0.40)	0.0016^*^
4	0.42(0.25 to 0.58)	< 0.001^*^
5	1.03(0.79 to 1.3)	< 0.001^*^
6	1.8(1.5 to 2.2)	< 0.001^*^
LOCS III (Cortical Cataract, vs grade 1)		
2	0.19(0.014 to 0.36)	0.034^*^
3	0.36(0.18 to 0.53)	< 0.001^*^
4	0.80(0.55 to 1.0)	< 0.001^*^
LOCS III (Posterior Cataract, vs grade 1)		
2	0.22(0.042 to 0.39)	0.015^*^
3	0.37(0.21 to 0.53)	< 0.001^*^
4	0.78(0.58 to 0.98)	< 0.001^*^
5	1.8(1.3 to 2.3)	< 0.001^*^

*CI* confidence interval

^*^Statistically significant *P* value

## Discussion

With advancement in surgical techniques and technologies, phacoemulsification in patients aged > 90 years has not been rare. However, visual outcomes postoperatively in this age group have remained unclear to date. We investigated a relatively large number of eyes in patients aged ≥90 years and elucidated how older age could affect VA outcomes after phacoemulsification compared to younger patients.

In the literature, several studies have evaluated effects of age on postoperative VA after cataract surgery, but not specifically after phacoemulsification. Some studies have shown that patients aged > 80 years had a significantly higher risk of failure to achieve postoperative VA better than 0.67 [9] or 6/12 [10]. Another study also indicated that patients aged > 90 years had a higher risk of poor VA outcome [11]. Another study of 37 patients aged ≥90 years also found that postoperative VA was worse with increasing age [12]. On the other hand, one study indicated that not age itself, but ocular comorbidities were responsible for the worse VA outcome in the very elderly population [6]. A limitation of these studies is that they were conducted a relatively long time ago, and their conclusions were based on surgeries performed before 2000. At that time, a significant number of cases had been treated with extracapsular cataract extraction (23% [11], 48% [9]) rather than phacoemulsification. A more recent report found no difference in postoperative VA between 31 eyes of patients aged ≥90 years and 70 eyes of patients aged < 90 years [13]. Other recent studies also have shown favorable VA outcomes after cataract surgery in patients aged ≥90 years, though without comparison to younger patients [14, 15]. Another limitation is that these previous studies have used postoperative VA as an outcome measure, but not improvement in postoperative VA. In our study both pre- and postoperative VA was significantly worse in the older patients as indicated in previous literature; however, the postoperative VA improvement was equally favorable in younger and older patients.

The features of cataract surgery in the very elderly include systemic/ocular comorbidities, impaired cognitive function, higher-grade cataract, risk of capsule rupture and dialysis, postoperative infection, and corneal edema. Our results suggested that when performed by experienced surgeons who are able to manage these risks using current techniques and devices, phacoemulsification cataract surgery is safe and effective even in patients aged ≥90 years.

In 2012, Mutoh et al. [13] reported similar rates of intraoperative complications in patients aged ≥90 years compared to those aged < 90 years, whereas in 2000, Berler et al. [16] reported significantly higher rates of intraoperative complications in patients aged ≥88 years compared to those aged < 88 years. In both studies, enrolled patients had undergone phacoemulsification. Our results are consistent with the more recent report by Mutoh et al. [13] and suggested that the development of surgical instruments and techniques in recent years has contributed to intraoperative safety for the very elderly patients undergoing phacoemulsification.

Postoperative complications in the very elderly compared to younger patients have not been clear in the literature. In 2004, Syam et al. [15] reported on postoperative complications after cataract surgery in patients aged > 96 years. In that study, phacoemulsification was performed in 30 eyes and extracapsular extraction in four, and the rate of postoperative complications was 11.8% (postoperative uveitis in two eyes and incarceration of iris paracentesis wound in two). They concluded that cataract surgery can be performed even in the very elderly. In our study, there was no statistical difference in the frequency of postoperative complications between younger and older patients (5.9% vs. 5.1%: *P* = 0.8). However, it should be noted that very elderly patients often needed help from family members for postoperative care, including eye drops and follow-up visits.

In our study, a history of DM significantly affected BCVA improvement at 3 months postoperatively, which is consistent with previous findings [11]. Other studies also indicated that diabetic patients without diabetic retinopathy have a thicker macula after cataract surgery [17, 18]. The increase in macular thickness is considered to occur 3 to 6 months after cataract surgery, which might explain why we found the effect of DM history on VA improvement at 3 months but not at 1 month postoperatively. In addition, another study reported that after cataract surgery, the mean interval between surgery and the first recording of postoperative macular edema was 39.5 days, and this was found in 4% of diabetic patients [19].

A limitation of our study was the retrospective nature based on data from a single hospital. All surgeries were performed by experienced surgeons, and our conclusions might not be true for less experienced surgeons. Relatively short follow-up duration might be another limitation, and extended follow-up might have clarified more post-surgical complications including posterior capsule opacity and lens dislocation. In addition, VA was the outcome measures in our study; however, other outcome measures, including contrast sensitivity and quality of life, also would be important for future studies.

## Conclusions

The present study has addressed for the first time BCVA improvement in the very elderly (i.e., ≥90 years old) after phacoemulsification cataract surgery, and our multivariate analyses have indicated its similar effectiveness and safety to younger patients.

## Abbreviations

BCVA: Best-corrected VA
BRVO: Branch retinal vein occlusion
CRVO: Central retinal vein occlusion
DM: Diabetes mellitus
HT: Hypertension
IOL: Intraocular lens
logMAR: Logarithm of the minimum angle of resolution
SD: Standard deviation
SE: Standard error
VA: Visual acuity
